# rTMS investigation of resistant Obsessive-Compulsive Related Disorders: Efficacy of targeting the reward system

**DOI:** 10.3389/fpsyt.2022.1035469

**Published:** 2023-02-03

**Authors:** Michele Di Ponzio, Nikos Makris, Carlotta Tenerini, Eleonora Grassi, Samuele Ragone, Stefano Pallanti

**Affiliations:** ^1^Institute for Neuroscience, Florence, Italy; ^2^Department of Psychiatry, Massachusetts General Hospital, Harvard Medical School, Boston, MA, United States; ^3^Department of Psychiatry, Center for Morphometric Analysis, A. A. Martinos Center for Biomedical Imaging, Harvard Medical School, Boston, MA, United States; ^4^Department of Neurology, Center for Morphometric Analysis, A. A. Martinos Center for Biomedical Imaging, Harvard Medical School, Boston, MA, United States; ^5^Department of Anatomy and Neurobiology, Boston University Medical School, Boston, MA, United States; ^6^Department of Psychiatry and Behavioral Science, Albert Einstein College of Medicine, Bronx, NY, United States

**Keywords:** rTMS (repetitive transcranial magnetic stimulation), Obsessive-Compulsive Related Disorders, OCD (obsessive-compulsive disorder), reward system, brain plasticity, brain stimulation, behavioral addictions

## Abstract

**Introduction:**

Repetitive Transcranial Magnetic Stimulation (rTMS) is not only a therapeutic option but also an investigational tool to explore circuits and subjective dimensions in pathological conditions. Obsessive-Compulsive Related Disorders (OCRDs) shared similarities with Substance Use Disorder (SUD), suggesting the involvement of the reward system. This study aimed to verify the efficacy of targeting the reward system with rTMS in OCRDs.

**Methods:**

Patients with trichotillomania, hoarding disorder and skin picking disorder were treated with rTMS over the left DorsoLateral PreFrontal Cortex (DLPFC) at 15 Hz, targeting the reward system via the connection with the nucleus accumbens and the ventral tegmental area. All patients were administered with psychometric scales assessing depression symptoms and severity of OCRDs symptoms at the baseline, at the end of the treatment and a 1-month follow-up.

**Results:**

Analysis of the results showed a reduction in symptom severity at the end of the treatment in all three groups (*p* < 0.0001) as well as a reduction in depression symptoms (*p* < 0.01). Improvements at 1-month follow-up were maintained only in younger patients. Indeed, when changes in scores at the follow-up were analyzed separately for younger (<30 years) and older patients (>60 years), the elderly showed again an increase in symptoms severity, suggesting that the stability of TMS effects over time reduces with age, possibly as an effect of age-related reduction in brain plasticity.

**Discussion:**

This study adopted with promising results a protocol (15 Hz over the left DLPFC) targeting the reward system, typically employed in addictions. These results can be in line with the view of OCRDs as behavioral addictions, suggesting the implication of common circuits, such as the reward system, in the mechanisms at the basis of these disorders.

## 1. Introduction

The Diagnostic and Statistical Manual-Fifth Edition [DSM-5; (1)] introduced the new diagnostic category of Obsessive-Compulsive and Related Disorders (OCRDs). It comprises trichotillomania (TTM; hair-pulling disorder), excoriation disorder (skin picking; SPD), obsessive-compulsive disorder (OCD), body dysmorphic disorder (BDD), and hoarding disorder (HD). Obsessions (repeated, upsetting, intrusive thoughts, visions, or desires) and compulsions (ritualized acts performed to relieve discomfort from obsessions) are the key symptoms of OCD (1). HD refers to the difficult in discarding, also worthless, possessions (1). Recurring hair pulling, which causes hair loss, is a defining feature of TTM (1). SP entails regular skin picking, which causes lesions (1).

All these disorders share compulsive behaviors as a cardinal feature, which are also typical of addictions (2). Based on this and other analogies, OCD has been proposed to be considered a behavioral addiction (3). Furthermore, an addiction model of TTM (4) and of SPD (5) has been proposed mainly based on similar clinical manifestations, including compulsivity, diminished inhibitory control, urge or craving state before the engagement in the hair pulling and the hedonic quality of performing hair pulling or skin picking. Furthermore, all compulsive behaviors indicate impaired reward processing, lack of inhibitory control, and cognitive inflexibility (2). Patients with OCD as well as with SPD and TTM (6) showed impaired motor and cognitive inhibitory mechanisms, suggesting impairment of frontostriatal circuitries which regulate inhibitory control (7). At the same time, reward processing dysfunction, which is one of the main feature of addictions (8), has been implicated in the etiology and sustention of SPD and TTM (9), suggesting that the intense craving and pleasure experienced during the behavior could be the result of abnormal brain reward processing (10).

Repetitive Transcranial Magnetic Stimulation (rTMS) has emerged as a valid therapeutic option for the treatment of OCD. Furthermore, its application might work as an investigational tool exploring circuits and subjective dimensions involved in the impulsive-compulsive phenomena. Mainly, four brain areas have been the different targets of rTMS in OCD, as emerged from a literature review (11): the DorsoLateral PreFrontal Cortex (DLPFC), the Supplementary Motor Area (SMA), the OrbitoFrontal Cortex (OFC) and the Anterior Cingulate Cortex (ACC). Positive outcomes have been reported for all the aforementioned targets. This evidence highlights the heterogeneity of OCD. In the specific case of OCRDs, only two studies have reported the effect of rTMS. One study was a case series report (12), in which patients with TTM were treated with low-frequency rTMS over the pre-SMA. Then, a prospective study failed to report the effects of rTMS over the pre-SMA in SPD (13). Concerning hoarding, only one case study reported the efficacy of prefrontal direct current stimulation (14). At our knowledge, no study investigated the effect of TMS in hoarding. Furthermore, no study specifically targeted DLPFC in OCRD, although encouraging results have been shown in OCD both with TMS (11) and direct current stimulation (15, 16). However, controversial results emerged concerning the optimal frequency of stimulation. Different studies have chosen to treat OCD patients with rTMS over the left DLPFC at 10 hz or 20 Hz (17–20). Then, rTMS over left DLPFC at 15 Hz has been previously shown to be effective in addiction to reducing craving and compulsive behaviors (21, 22), due to its involvement in reward circuitries (23, 24). No FDA-approved treatment for OCRD exists.

In light of the addiction hypothesis of OCRD and given the negative results of pre-SMA stimulations, we have proposed rTMS over the left DLPFC at 15 Hz for the treatment of patients with OCRD in our center (Istituto di Neuroscienze, Florence, Italy). In our center, we use rTMS for different disorders and all the data are collected in our databases. Herein, data are reported and analyzed retrospectively, to examine the clinical profile of patients with TTM, SPD, and HD treated with rTMS at 15 Hz over the left DLPFC before, after treatment and at 1-month follow-up, with the aim also to propose the possibility that OCRDs are linked with addictions. Moreover, the potential effect of age was analyzed.

## 2. Materials and methods

### 2.1. Participants

In this retrospective study, clinical data of patients with a diagnosis of Obsessive-Compulsive Related Disorders (SPD, TTM, and HD) according to DSM-5 criteria were extracted from databases containing information on patients of the psychiatric clinic at the Istituto di Neuroscience, Florence (Italy). Patients’ age ranged from 16 to 76 years old. All patients had a history of cognitive-behavioral therapy, but no one was under psychotherapy while treated with TMS. Moreover, all patients were resistant to treatment, based on the operational definition by Pallanti and Quercioli (25). It is important to mention that the database used for the analysis contained only the data of patients who accepted treatment among all the ones to which was proposed during the normal clinical practice: 41 accepted out of 60 to which was proposed (information obtained from the clinic’s internal system). The reason for the ones who did not accept to start the protocol, despite the indication for treatment with rTMS, were the choice for other types of medications or their inability (for personal reasons) to follow the entire cycle of TMS. rTMS was added to ongoing pharmacological treatments. All patients were treated stably for 2 months with Selective Serotonin Reuptake Inhibitors (SSRIs) at a fluoxetine equivalent dosage of 30 mg. Demographical data are reported in Table 1 (see also Supplementary Table 1). After the complete description of the study to participants, written informed consent was obtained from each one for the inclusion of their data in this study.

**TABLE 1:** Descriptive statistics for each group, reporting sample size, age mean and standard deviation, and male/female ratios.

Group	Sample size	Age	Gender	Comorbidities
Hoarding	14	48.7 (18.5)	5 M, 9 F	5 MDD, 2 ADHD, 1 SUD, 4 GAD
Skin picking	13	43.5 (20)	4 M, 9 F	3 SUD, 7 MDD, 2 ADHD, 3 GAD, 1 bipolar disorder
Trichotillomania	14	41 (19.5)	2 M, 12 F	7 MDD, 2 ADHD, 4 GAD, 1 bipolar disorder

MDD, major depressive disorder; ADHD, attention deficit hyperactivity disorder; SUD, substance use disorder; GAD, generalized anxiety disorder.

### 2.2. Procedure

Repetitive Transcranial Magnetic Stimulation was administered with the Magstim Rapid Stimulator (Magstim Company Ltd., Whitland, UK) using a 70-mm, 8-shaped coil. Stimulation parameters were 15 Hz, 2,400 pulses/day at 100% of resting motor threshold (MT), once a day, 6 days/week for 4 weeks (24 sessions total). Stimulation was applied on the left DLPFC, identified for each subject through neuronavigation. Resting MT was defined as the minimum magnetic flux needed to elicit a response in a resting target muscle (abductor pollicis brevis) in 5/10 trials using single-pulse TMS administered to the contralateral primary motor cortex.

### 2.3. Psychometric measures

Baseline assessments were performed before the first rTMS session and repeated at the end of the treatment. Follow-up assessments were performed 1 month after the end of the treatment. The assessment has been performed by a panel of trained raters but blind to the treatment administered.

The Massachusetts General Hospital Hair pulling Scale (MGH) (26) assesses the frequency, intensity, and distress of trichotillomania behavior. It consists of seven items with a maximum score of 28. A score between 0–7 refers to subclinical symptomatology, between 8–14 to mild symptomatology, between 15–21 to moderate symptomatology, and between 22–28 to severe symptomatology. Since the questionnaire was not available in the Italian, two independent native Italian speakers fluent in English translated the original scale into Italian. This translated version was then translated back into English by two separate native English speakers who were also fluent in Italian. No significant differences were found between the original and the newly translated version. The Cronbach’s alpha of the Italian version of the scale administered here was 0.89, indicating excellent internal consistency.

The Hoarding Rating Scale-Interview [HRS-I; (27)] is a 5-item semi-structured interview that assesses clutter, difficulty discarding, acquiring, distress, and impairment. Each item is rated on a 9-point scale from 0 to 8, and the item scores are summed to create a total score (range = 0–40). A score higher than 14 is associated with significant impairment in daily life due to difficulty discarding. The Italian version, validated by Faraci et al. (28) was used.

The Yale-Brown Obsessive Compulsive Scale Modified for Neurotic Excoriation (NE-YBOCS) is valid and reliable scale used to evaluate the severity of SPD. Responses to the 10 items were coded on a 4-point scale and summed to produce a composite score ranging from 0 to 40, with higher scores reflecting greater illness severity. Since the questionnaire was not available in the Italian, two independent native Italian speakers fluent in English translated the original scale into Italian. This translated version was then translated back into English by two separate native English speakers who were also fluent in Italian. No significant differences were found between the original and the newly translated version. The Cronbach’s alpha of the Italian version of the scale administered here was 0.92, indicating excellent internal consistency.

The Italian version of the Symptoms of Depression Questionnaire [SDQ; (29)] was used in this study. It is a 44-item, Likert-type, self-report scale developed for measuring symptom severity across several subtypes of depression. SDQ encloses five subscales, investigating the following dimensions: lassitude, mood, cognitive/social functioning; anxiety, agitation, anger and irritability; the desire to be dead; disruptions in sleep quality; changes in appetite and weight.

### 2.4. Statistical analysis

The baseline demographic and clinical characteristics of the sample were tabulated with descriptive statistics. Parametric (*t*-test) and non-parametric (Wilcoxon) tests were used according to variables’ distribution (tested with the Shapiro-Wilk test) to analyze changes in scores over time and to compare scores at the baseline between those who accepted to be treated with TMS and those who refused. A regression analysis (Pearson’s correlation) was used to test the effect of age and to verify whether the change in symptoms severity (score of each symptomatologic scale) was dependent to the change in SDQ scores between the pre- and post-treatment. For all statistical analyses, the alpha level of significance was set at 0.05. All the statistical analyses were performed using the statistical programming language R (version 4.0.5) (30).

## 3. Results

The study included 41 patients, which were dived into three groups based on the diagnosis. The SPD group consisted of 13 patients (9 females; mean age: 43.5; SD: 20). The TTM group consisted of 14 patients (12 females; mean age: 41; SD: 19.5). The HD group consisted of 14 patients (9 females: mean age: 48.7; SD: 18.5) (see Table 1). Scores statistics are reported in Table 2. For detailed score report, please see Supplementary Tables 2–4.

**TABLE 2:** Mean scores (and standard deviations) of the psychometrics scale are reported for each group at the pre- and post-treatment timepoints, as well as the percentage of score reduction after treatment.

Group	Scales	Pre-test	Post-test	Percentage of change	*p*-value	Effect size
Hoarding	HRS	26 (4.3)	12.4 (3.5)	52.4 (12.1)	<0.0001	3.61
	SDQ	136 (16.7)	108 (14.8)	20.4 (9.5)	<0.0001	1.87
Trichotillomania	MGH	21 (4.1)	9.1 (4.32)	58.2 (17.1)	<0.0001	3.52
	SDQ	130 (9.6)	107 (9.4)	17.1 (8.5)	<0.0001	1.78
Skin picking	NE-YBOCS	26.8 (6.15)	10.2 (4.86)	63 (13.8)	<0.0001	3.77
	SDQ	131 (14.8)	99.7 (11.9)	22 (6.5)	<0.0001	3.66

*P*-value and effect sizes of each comparison (*t*-test) between pre- and post-treatment scores are also reported. HRS, Hoarding Rating Scale; SDQ, symptoms of depression questionnaire; MGH, Massachusetts General Hospital Hair pulling Scale; NE-YBOCS, Neurotic Excoriations Yale-Brown Obsessive-Compulsive Scale.

Baseline scale measures were compared between the 41 patients who accepted to be treated and the ones who refused TMS treatment as well as age distribution, in order to verify whether there were differences between these two groups. No statistically significant differences were found.

As HRS scores in the HD group were normally distributed (verified through the Shapiro–Wilk test), a multiple paired *t*-test was used to determine whether there were differences in scores between pre-and post-treatment and between post-treatment and follow-up. All patients improved at the end of the treatment (Table 2), with a mean percentage of improvement of 52%. HRS scores before and after treatment were statistically different (*p* < 0.0001), while there was no statistically significant difference between post-treatment and follow-up scores.

As MGH scores in the TTM group were normally distributed (verified through the Shapiro–Wilk test), a multiple paired *t*-test was used to determine whether there were differences in scores between pre- and post-treatment and between post-treatment and follow-up. All patients improved at the end of the treatment (Table 2), with a mean percentage of improvement of 58%. MGH scores before and after treatment were statistically different (*p* < 0.0001), while there was no statistically significant difference between post-treatment and follow-up scores.

As NE-YBOCS scores in the SPD group were normally distributed (verified through the Shapiro–Wilk test), a multiple paired *t*-test was used to determine whether there were differences in scores between pre- and post-treatment and between post-treatment and follow-up. All patients improved at the end of the treatment (Table 3), with a mean percentage of improvement of 62%. NE-YBOCS scores before and after treatment were statistically different (*p* < 0.0001), while there was no statistically significant difference between post-treatment and follow-up scores.

**TABLE 3:** Demographical data (sample size, age mean and standard deviation) are here reported for each group (Hoarding, Trichotillomania, and Skin Picking) in the subgroups: older (>60 years of age) vs. younger adults (<35 years of age).

Group	Subgroup	Sample size	Age	Pre-test	Post-test	Follow-up
Hoarding	Old	6 (4 female)	66.7 (5.4)	27.2 (5.78)	12.5 (4.55)	16.3 (4.37)
	Young	5 (3 female)	28 (7.2)	24.6 (2.51)	13 (2.24)	12.2 (0.84)
Trichotillomania	Old	5 (3 female)	63.2 (2.3)	20 (3.8)	10.4 (4.3)	14 (4.36)
	Young	8 (8 female)	25.1 (5.8)	22.1 (4.3)	8.5 (4.7)	7.6 (3.6)
Skin picking	Old	5 (4 female)	63.6 (2.4)	27.8 (7.7)	11 (6.8)	16.4 (7.8)
	Young	6 (3 female)	23.5 (6.1)	28 (4.9)	10.3 (4)	9.17 (2.8)

Mean scores and standard deviations of psychometric scales are reported: Hoarding Rating Scale (HRS) for the hoarding group; Massachusetts General Hospital Hair pulling Scale (MGH) for the trichotillomania group; Neurotic Excoriation Yale-Brown Obsessive-Compulsive Scale for the Skin Picking Disorder.

As SDQ scores, for all groups, were normally distributed (verified through the Shapiro–Wilk test), a multiple paired *t*-test was used to determine whether there were differences in scores between pre-and post-treatment and between post-treatment and follow-up for each group. In the hoarding group, the mean reduction corresponded to 20%; in the TTM group, 17% and in the SPD group, 23%. SDQ scores before and after treatment were statistically different (*p* < 0.0001), while there was no statistically significant difference between post-treatment and follow-up scores for each group.

Linear regression was performed to verify a potential age effect in all three groups, due to the wide range of ages in this sample. Linear regression was performed before between the score difference between pre- and post-treatment and age, then between the score difference between the post-treatment and the follow-up. Regarding the HD group and TTM group, no age effect was found in the score difference between pre- and post-treatment. While the correlation between age and the difference in scores between post-treatment and follow-up scores was significant with a *p*-value of < 0.01. Regarding the SPD group, no age effect was found in the score difference between pre- and post-treatment. While the correlation between age and the difference in scores between post-treatment and follow-up scores was significant with a *p*-value of < 0.001.

To further investigate the effect of age, given the results obtained with the correlation, participants in each group were divided into two subgroups based on their age. The young adult group included patients younger than 35 years of age and the older adults group included patients older than 60 years of age (see Table 3).

**FIGURE 1:**
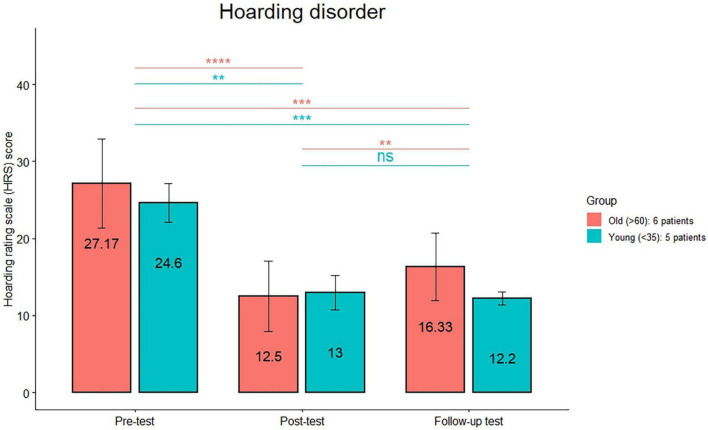
Hoarding Rating Scale mean scores are reported for the young and old subgroups in the hoarding group at the three timepoints (pre-treatment, post-treatment, and 1 month follow-up). ****p < 0.0001; ***p < 0.001; **p < 0.01; n.s.: not significant.

**FIGURE 2:**
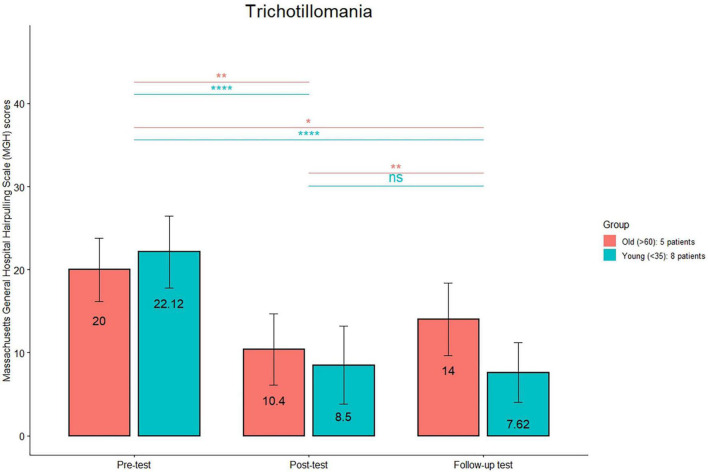
Massachusetts General Hospital Hair pulling Scale (MGH) mean scores are reported for the young and old subgroups in the trichotillomania group at the three timepoints (pre-treatment, post-treatment, and 1 month follow-up). ****p < 0.0001; **p < 0.01; *p < 0.05; n.s.: not significant.

**FIGURE 3:**
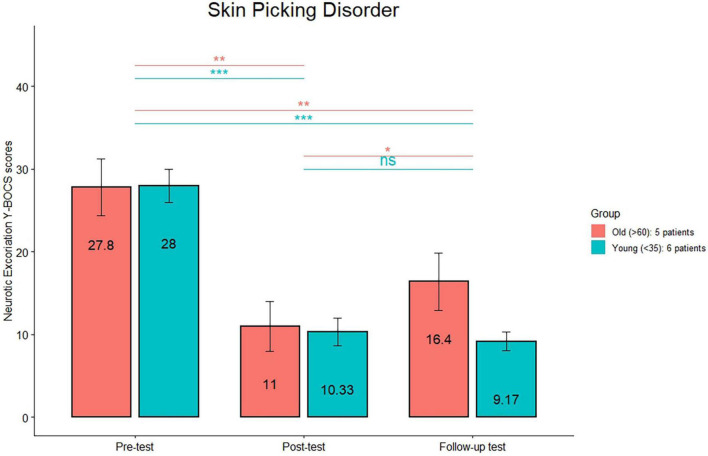
Neurotic Excoriations Yale-Brown Obsessive-Compulsive Scale mean scores are reported for the young and old subgroups in the trichotillomania group at the three timepoints (pre-treatment, post-treatment, and 1 month follow-up). ***p < 0.0001; **p < 0.01; *p < 0.05; n.s.: not significant.

The comparison (see Figure 1) between the HRS scores between post-treatment and follow-up was significant only in the old group (*p* < 0.01). The comparison (see Figure 2) between the MGH scores between post-treatment and follow-up was significant only in the old group (*p* < 0.01). The comparison (see Figure 3) between the NE-YBOCS scores between post-treatment and follow-up was significant only in the old group (*p* < 0.05).

In order to verify whether the improvement in symptoms severity was due to the improvement in comorbid depression, a linear regression was used to assess whether there was a relationship between the change between the pre and post treatment in SDQ scores and the change in HRS, NE-YBOCS, and MGH scores. No significant results were obtained for any measure. Importantly, no side effects were reported by the patients.

## 4. Discussion

This is the first study to report the effects of high-frequency (15 Hz) rTMS over the left DLPFC in OCRD. The main finding of this retrospective study was the positive response of OCRD patients to treatment with a reduction of symptoms severity of more than 35%, which is the conventional threshold to discriminate between respondents and not respondents to treatment in OCD (25). Moreover, an improvement in depression symptoms was also observed. Therefore, given the lack of approved treatments for OCRD and the promising results here reported, this study suggests that this protocol can be a possible treatment for OCRD, that could open a new therapeutic pathway as already occurred in Substance Use Disorder.

There is no consensus on the optimal target and protocol of TMS in OCD. Low-frequency TMS of the supplementary motor cortex has been shown to alleviate OCD symptoms in many but not all studies (11). Studies investigating high-frequency (10 Hz) stimulation over the DLPFC also showed controversial results (11), as well as studies adopting 20 hz frequency stimulations (19, 20). Recently, Khurshid (31) hypothesized that high-frequency rTMS of pre-SMA can reduce OCD symptoms. Here, instead, we tested the efficacy of high-frequency (15 hz) stimulation over left DLPFC. High-frequency 15 HZ rTMS over DLPFC is a treatment for addictions, such as cocaine (21, 22), due to the modulation of activity in subcortical reward circuitry involving the dopaminergic midbrain ventral tegmental area and nucleus accumbens (23, 32). One study provided strong evidence that stimulation of left DLPFC influences the ACC (33), which has a specific role in reward decision-making (34). ACC shows alterations in OCD and also in skin picking (35). Therefore, the positive outcomes here reported in OCRD suggested an implication of reward circuits. It can be hypothesized that, given the positive outcomes of a protocol usually employed for addictions, our results are consistent with the emerging view of OCD as a behavioral addiction (36), a conclusion that could be spread to the entire spectrum. As a matter of fact, people with OCRDs have an high comorbidity rates of addiction (37) and are more likely than controls to have first-degree relatives with Substance Use Disorder (38). Furthermore, double-blind placebo-controlled trials have shown that pharmacological treatments targeting the reward processing by modulating glutamate and dopamine are effective in OCRDs (39). Moreover, the involvement of reward circuitry in OCRDs has been supported by a fMRI study which found alterations in reward circuitry (9).

The hypothesis is that there is a continuum that goes from impulsivity through compulsivity to addiction and the transition to addiction involved a shift from hyperactivation of the ventral striatum to the dorsal striatum (40) and also a progressive loss of top-down, executive control resulting from a loss of PFC and cingulate cortex function (36).

Concerning the controversial results of HF l-DLPFC in OCD (11), it is reasonable to believe that since OCD is an heterogeneous disorder, the individuals who benefited the most from that treatment were the one with features more similar to OCRD. In this sense, they could be clustered into a “reward deficiency group,” adopting the terminology by Lochner et al. (41). Again, this could result in a different neurocircuitry involvement, with a preferential involvement of the complex DLPFC/ACC in “reward deficiency group” and a pre-SMA involvement in the “impulsive” group. Furthermore, it could be that these two groups experience differently their symptoms, with a different level of awareness. The same explanation could apply to the differences in outcomes between the TTM and SPD groups and HD group. The last one showed a percentage of improvement inferior to the ones obtained in the other two groups. Reasonably, HD could be characterized by features, such as the attentional component (42), that may not match perfectly the ones of the “reward deficiency group.”

These results are in line with a multidimensional perspective of OCD (43), which lies in the middle between a lumping and a splitting view. According to the lumping view, OCD is a unitary disorder; while, the splitting perspective claims that different subtypes of OCD exist which all represent different disorders, with different causes and different treatments. But, according to an intermediate view, OCD is a spectrum of overlapping disorders, which have their specificities but share also some similarities. Accordingly, they can share the same neural substrates, such as DLPFC alterations. Although this speculation is beyond the actual implications of this study, the fact that the previous study (13) failed to replicate for SPD the same results that have been obtained for OCD and the fact that instead the study here presented replicated them for DLPFC can mean that the common link could be an alteration of DLPFC. Indeed, considering that compulsive behaviors are a cardinal feature of the OCD spectrum, recently, Fremont et al. (44) have found that reductions in the left DLPFC were associated with the development of compulsive behaviors not accompanied by obsessions. Coherently, in TTM and SPD compulsions are not necessarily triggered by obsessional thoughts, as they are not in the DSM–5 diagnostic criteria (1).

Regarding the other results of this study, no difference was found between scores at the end of the treatment and 1-month follow-up, suggesting that the rTMS effect can last beyond the end of the treatment. Interestingly, when patients were divided into two groups based on their age, differences emerged concerning the maintenance of beneficial effects of rTMS at the follow-up. Indeed, results showed that in older adults symptoms severity at the follow-up worsened again, while in young adults the results were stable over time. Reasonably, this result can be a consequence of a reduction of plasticity in older brains. This result is coherent with other findings (45). For example, in a study with an adult age ranging from 19 to 81 years, Freitas et al. (46) found the duration and magnitude of corticospinal excitability modulation by rTMS were inversely and significantly correlated with age. Furthermore, a recent study by D’Urso et al. (47) found an inverse correlation between age and clinical response to TMS treatment in resistant-depression. These data provide direct experimental evidence that, in humans, long-term plasticity becomes increasingly less efficient with advancing age.

The present study has some limitations, including its retrospective nature, the lack of a control group, addressing the potential placebo effect (although blind raters were involved to minimize the confounding effects) and the low sample size. Furthermore, although the inclusion of a follow-up assessment and the stability of effects in younger participants, it is a relative short term follow-up, considering that Aydin et al. (12) found a re-worsening of symptoms in TTM patients after TMS at a 3-month follow-up. In this sense, we believe that, based on unpublished data in our possession, a monthly follow-up booster session could be helpful in the stability of the effects over time. Furthermore, right DLPFC has also been implicated in reward functioning. We cannot conclude about the potential effect of targeting right DLPFC at high-frequency in OCRDs. Future research should overcome these limitations and should prospectively analyze the effects of rTMS in OCRD over the DLPFC and should also investigate the neuroimaging correlates, in order to corroborate the hypotheses here formulated. Being a naturalistic study, it was not possible to control for comorbidities, such as ADHD, which appeared to be frequent in our sample, as reported in the methods section. ADHD is characterized by frontal dysfunctions, but, as Cardullo et al. (48) reported, ADHD comorbidity with psychiatric disorders did not interfere with rTMS application.

Recognition that neural networks are interconnected and communicate at different levels can facilitate a better understanding of the neurobiological concepts related to psychiatric disorders and also of treatment with rTMS. In the future, targets for rTMS should be no more anatomical but should look at the functional connections of the target. In this sense, the target should be chosen depending on its connectivity (49). Our study points in this direction. Indeed, its positive outcomes acquire sense only by looking at the connections and at the neural networks in which the left DLPFC is involved.

## Data availability statement

The raw data supporting the conclusions of this article will be made available by the authors, without undue reservation.

## Ethics statement

Ethical review and approval was not required for the study on human participants in accordance with the local legislation and institutional requirements. The patients/participants provided their written informed consent to participate in this study. Written informed consent was obtained from each participant for the inclusion of their potentially identifiable data in this study.

## Author contributions

SP and MD: conceptualization. MD: formal analysis. SP, EG, and MD: investigation. EG, CT, and SR: data curation. SP, MD, EG, CT, SR, and NM: writing—original draft preparation. SP and NM: supervision. All authors contributed to the article and approved the submitted version.
