# Supplementary material for: rTMS investigation of resistant Obsessive-Compulsive Related Disorders: Efficacy of targeting the reward system
Source: Front Psychiatry. 2023 Feb 3;13:1035469. doi: 10.3389/fpsyt.2022.1035469 (PMC9937025; doi:10.3389/fpsyt.2022.1035469)
Supplement: Supplementary file 1 [file Table_1.docx]

Supplementary Material

- 1. **Supplementary Tables**

Supplementary Table 1. Demographical data of the patients (MDD: Major Depressive Disorder; ADHD, Attention Deficit Hyperactivity Disorder; SUD, Substance Use Disorder; GAD, Generalized Anxiety Disorder).

| **Id** | **Gender** | **Age** | **Diagnosis** | **Comorbidity** |
| --- | --- | --- | --- | --- |
| 1 | F | 17 | Hoarding | ADHD; SUD |
| 2 | M | 25 | Hoarding | MDD |
| 3 | F | 30 | Hoarding |  |
| 4 | M | 34 | Hoarding |  |
| 5 | F | 34 | Hoarding | MDD |
| 6 | F | 61 | Hoarding | MDD |
| 7 | M | 43 | Hoarding | GAD |
| 8 | F | 48 | Hoarding | GAD; MDD |
| 9 | F | 51 | Hoarding |  |
| 10 | F | 68 | Hoarding | ADHD |
| 11 | F | 60 | Hoarding |  |
| 12 | F | 67 | Hoarding | GAD |
| 13 | M | 74 | Hoarding | GAD |
| 14 | M | 70 | Hoarding | MDD |
| 15 | F | 16 | skin picking | SUD; MDD |
| 16 | M | 18 | skin picking | SUD; ADHD |
| 17 | F | 21 | skin picking | ADHD; SUD |
| 18 | M | 26 | skin picking | MDD |
| 19 | F | 30 | skin picking | MDD; GAD |
| 20 | M | 30 | skin picking | MDD |
| 21 | F | 61 | skin picking | MDD; GAD |
| 22 | F | 65 | skin picking |  |
| 23 | F | 51 | skin picking | MDD |
| 24 | F | 55 | skin picking | MDD |
| 25 | M | 62 | skin picking | Bipolar Disorder |
| 26 | F | 67 | skin picking |  |
| 27 | F | 63 | skin picking | GAD |
| 28 | F | 17 | trichotillomania | MDD |
| 29 | F | 18 | trichotillomania | ADHD |
| 30 | F | 22 | trichotillomania | MDD |
| 31 | F | 23 | trichotillomania | GAD |
| 32 | F | 30 | trichotillomania | Bipolar Disorder |
| 33 | F | 30 | trichotillomania | GAD; MDD |
| 34 | F | 30 | trichotillomania |  |
| 35 | F | 31 | trichotillomania | ADHD |
| 36 | F | 66 | trichotillomania | MDD; GAD |
| 37 | F | 56 | trichotillomania | MDD |
| 38 | F | 60 | trichotillomania |  |
| 39 | F | 62 | trichotillomania | MDD |
| 40 | M | 64 | trichotillomania | MDD |
| 41 | M | 64 | trichotillomania | GAD |

Table 2 Percentage of psychometric scales scores reduction in the hoarding group

| ***Patient*** | ***HRS*** | | | ***SDQ*** | | |
| --- | --- | --- | --- | --- | --- | --- |
|  | ***Pre-test*** | ***Post-test*** | ***Follow-up test*** | ***Pre-test*** | ***Post-test*** | ***Follow-up test*** |
| 1 | 24 | 12 | 12 | 140 | 110 | 112 |
| 2 | 25 | 10 | 11 | 142 | 121 | 140 |
| 3 | 28 | 16 | 13 | 138 | 98 | 110 |
| 4 | 21 | 14 | 12 | 115 | 105 | 103 |
| 5 | 25 | 13 | 13 | 108 | 83 | 85 |
| 6 | 29 | 13 | 17 | 127 | 119 | 115 |
| 7 | 22 | 15 | 12 | 155 | 129 | 121 |
| 8 | 27 | 8 | 8 | 166 | 104 | 107 |
| 9 | 29 | 10 | 12 | 131 | 96 | 98 |
| 10 | 22 | 7 | 12 | 139 | 91 | 91 |
| 11 | 19 | 7 | 10 | 111 | 91 | 100 |
| 12 | 35 | 17 | 21 | 152 | 113 | 105 |
| 13 | 30 | 17 | 19 | 146 | 131 | 128 |
| 14 | 28 | 14 | 19 | 136 | 118 | 114 |

Table 3 Percentage of psychometic scales scores improvement in the TTM group

| ***Patient*** | ***MGH*** | | | ***SDQ*** | | |
| --- | --- | --- | --- | --- | --- | --- |
|  | ***Pre-test*** | ***Post-test*** | ***Follow-up test*** | ***Pre-test*** | ***Post-test*** | ***Follow-up test*** |
| 1 | 24 | 12 | 12 | 133 | 123 | 130 |
| 2 | 26 | 14 | 10 | 129 | 114 | 113 |
| 3 | 22 | 7 | 7 | 156 | 103 | 97 |
| 4 | 12 | 2 | 4 | 125 | 100 | 110 |
| 5 | 23 | 8 | 6 | 127 | 93 | 100 |
| 6 | 22 | 5 | 5 | 114 | 101 | 111 |
| 7 | 23 | 5 | 4 | 129 | 113 | 112 |
| 8 | 25 | 15 | 13 | 130 | 100 | 98 |
| 9 | 15 | 3 | 7 | 119 | 110 | 106 |
| 10 | 17 | 7 | 5 | 138 | 121 | 122 |
| 11 | 25 | 12 | 15 | 128 | 115 | 116 |
| 12 | 18 | 11 | 13 | 125 | 111 | 112 |
| 13 | 20 | 12 | 17 | 134 | 99 | 102 |
| 14 | 22 | 14 | 18 | 128 | 96 | 85 |

Table 4 Percentage of psychometic scales scores improvement in the SP group

| ***Patient*** | ***NE-YBOCS*** | | | ***SDQ*** | | |
| --- | --- | --- | --- | --- | --- | --- |
|  | ***Pre-test*** | ***Post-test*** | ***Follow-up test*** | ***Pre-test*** | ***Post-test*** | ***Follow-up test*** |
| 1 | 31 | 17 | 13 | 152 | 125 | 122 |
| 2 | 32 | 8 | 8 | 137 | 110 | 111 |
| 3 | 23 | 10 | 8 | 114 | 101 | 114 |
| 4 | 23 | 10 | 10 | 121 | 92 | 97 |
| 5 | 34 | 12 | 11 | 133 | 96 | 82 |
| 6 | 25 | 5 | 5 | 140 | 90 | 81 |
| 7 | 23 | 5 | 9 | 110 | 89 | 99 |
| 8 | 20 | 3 | 8 | 113 | 83 | 85 |
| 9 | 24 | 7 | 8 | 118 | 87 | 87 |
| 10 | 18 | 8 | 7 | 133 | 99 | 101 |
| 11 | 35 | 19 | 23 | 145 | 111 | 115 |
| 12 | 37 | 15 | 25 | 154 | 105 | 102 |
| 13 | 24 | 13 | 17 | 132 | 108 | 118 |
